# Risk Assessment by Pre-surgical Tractography in Left Hemisphere Low-Grade Gliomas

**DOI:** 10.3389/fneur.2021.648432

**Published:** 2021-02-15

**Authors:** Tamara Ius, Teresa Somma, Cinzia Baiano, Ilaria Guarracino, Giada Pauletto, Annacarmen Nilo, Marta Maieron, Francesca Palese, Miran Skrap, Barbara Tomasino

**Affiliations:** ^1^Neurosurgery Unit, Department of Neurosciences, Santa Maria della Misericordia University Hospital, Udine, Italy; ^2^Division of Neurosurgery, Department of Neurosciences, Reproductive and Odontostomatological Sciences, Università degli Studi di Napoli Federico II, Naples, Italy; ^3^Scientific Institute, Istituto di Ricovero e Cura a Carattere Scientifico (IRCCS) E. Medea, Pordenone, Italy; ^4^Neurology Unit, Department of Neurosciences, Santa Maria della Misericordia University Hospital, Udine, Italy; ^5^Clinical Neurology Unit, Department of Neurosciences, Santa Maria della Misericordia University Hospital, Udine, Italy; ^6^Medical Physics, Santa Maria della Misericordia University Hospital, Udine, Italy; ^7^Medical Area Department, University hospital, Udine, Italy

**Keywords:** diffusion tensor imaging analysis, intraoperative electric stimulation, low-grade gliomas, inferior fronto-occipital fasciculus, arcuate fasciculus

## Abstract

**Background:** Tracking the white matter principal tracts is routinely typically included during the pre-surgery planning examinations and has revealed to limit functional resection of low-grade gliomas (LGGs) in eloquent areas.

**Objective:** We examined the integrity of the Superior Longitudinal Fasciculus (SLF) and Inferior Fronto-Occipital Fasciculus (IFOF), both known to be part of the language-related network in patients with LGGs involving the temporo-insular cortex. In a comparative approach, we contrasted the main quantitative fiber tracking values in the tumoral (T) and healthy (H) hemispheres to test whether or not this ratio could discriminate amongst patients with different post-operative outcomes.

**Methods:** Twenty-six patients with LGGs were included. We obtained quantitative fiber tracking values in the tumoral and healthy hemispheres and calculated the ratio (H_IFOF_–T_IFOF_)/H_IFOF_ and the ratio (H_SLF_–T_SLF_)/H_SLF_ on the number of streamlines. We analyzed how these values varied between patients with and without post-operative neurological outcomes and between patients with different post-operative Engel classes.

**Results:** The ratio for both IFOF and SLF significantly differed between patient with and without post-operative neurological language deficits. No associations were found between white matter structural changes and post-operative seizure outcomes.

**Conclusions:** Calculating the ratio on the number of streamlines and fractional anisotropy between the tumoral and the healthy hemispheres resulted to be a useful approach, which can prove to be useful during the pre-operative planning examination, as it gives a glimpse on the potential clinical outcomes in patients with LGGs involving the left temporo-insular cortex.

## Introduction

Low-grade gliomas (LGGs) present a surgical challenge because of the poorly defined tumoral borders and the infiltration of white matter tracts (1, 2). Maximal safe resection is the cornerstone of LGG management, in order to provide an optimal survival benefit and preserving the quality of life (3, 4). Pre-operative risk predictions based on anatomical data are insufficient in patients with language area-related LGG, mainly considering the higher inter-individual variance of functional language anatomy (2, 5). It is thus of utmost importance to develop a reliable non-invasive pre-operative method to estimate the risk of post-operative deficits in the clinical management of these patients.

Despite the inherent limitations associated with imaging reconstruction algorithms, DTI (Diffusion Tensor Imaging) has recently been included in the presurgical glioma workup to aid in the mapping of functional pathways and to prevent extensive damage associated with radical resection (3, 6). Although DTI currently represents the only way to investigate white matter in humans *in vivo* (7), providing a non-invasive and feasible method for evaluating changes in the main language pathways, its pre-operative predictive role to estimate the risk of post-operative deficits is poorly investigated.

The aim of our study is to use pre-operative DTI data to examine changes in the main pathways of the left temporal lobe, in patients with LGG and investigating relative differences within the Superior Longitudinal Fasciculus (SLF) and Inferior Fronto-Occipital Fasciculus (IFOF).

In a previous study (8), we performed such comparison in a group of thirty-seven patients with LGGs involving the corticospinal tract. In particular, a biomarker derived from DTI differences between healthy and tumoral hemisphere was explored. The study showed that patients who had a certain pre-operative index (<0.22), calculated by comparing the healthy and the impaired hemisphere, had a significantly lower risk of developing transient post-operative deficits (8).

In this current investigation, we applied the same analytic process on the SLF and IFOF in a consecutive series of patients with LGG in the temporo-insular cortex. In a comparative approach, we contrasted the main quantitative fiber tracking values in the tumoral (T) and healthy (H) hemispheres to test whether or not this ratio could discriminate among patients with different post-operative outcomes. In addition, as secondary endpoint, we also analyzed the potential relationships between the structural white matter (WM) changes induced by LGG infiltrative growing and post-operative seizure outcomes.

## Materials and Methods

### Study Population

We retrospectively reviewed a consecutive series of patients operated for LGG in the left hemisphere between 2012 and 2018.

Twenty-six patients met the strict following inclusion criteria:

- Involvement of the temporo-insular cortex.
- Age ≥ 18 years.
- No previous surgery.
- No pre-operative chemo- or radiotherapy.
- At least 18 months of follow-up.
- Availability of pre-operative 3-Tesla MRI including DTI.
- Objective evaluation of pre-operative tumor volume and extent of resection (EOR) on MRI images in DICOM format based on T2-weighted MRI sequences.
- Revision of histopathological specimens by using the 2016 WHO Classification of Tumors of the Central Nervous System (9).
- All patients underwent awake surgery, brain mapping, neurophysiological monitoring and intraoperative real-time neuropsychological testing (RTNT).

Needle biopsies were excluded from the study.

Medical diaries were reviewed for history of tumor- related epilepsy, seizure frequency and ictal semiology, number and type of anti-seizure medications (ASMs).

The 2017 ILAE classification was applied to classify seizures (10). For statistical analysis, seizures were dichotomized, according to ictal semeiology, in motor (tonic, atonic, clonic, myoclonic, and hypermotor) and non-motor (sensory, autonomic, emotional, and cognitive) seizures. After surgery, seizures outcome was defined following the Engel Classification of Seizures and dichotomized as Engel Class Ia (completely seizure free) vs. all the others (11). Engel Class at 1-year follow-up was used for the analysis.

The local Ethics Committee, Comitato Etico Unico Regionale del Friuli Venezia Giulia, approved this investigation (protocol N.0036567/P/GEN/EGAS, ID study 2540). Considering the retrospective nature of the study, written consent to participate in the study was not applicable. Written informed consent was obtained for surgery.

### Intraoperative Surgical Protocol

The surgical procedures were conducted under cortical and subcortical white matter brain mapping, according to the intraoperative technique previously described. In addition to Direct Electrical Stimulation (DES), real-time neuropsychological testing (RTNT) was applied during Awake Surgery (AS) (1, 12).

### Histological and Molecular Analysis

All histological samples were reviewed according to the 2016 World Health Organization (WHO) classification (9). Molecular markers were evaluated as previously described (13).

### MRI Data

#### Acquisition

MRI examination, anatomical and DTI images were performed at a 3T MR system (Achieva, Philips medical system) using an SENSE eight-element phased array head coil. The images included an high-resolution T2-weighted (TR/TE, = 2500/368.328 ms; FOV = 240 mm; 190 sagittal slices; voxel size, 1 × 1 × 1 mm) and a post-gadolinium contrast T1-weighted anatomic images (TR/TE = 8.100/3.707 ms; FOV = 240 mm; 190 sagittal slices; voxel size =1 × 1 × 1 mm) both optimized for the standard pre-operative clinical protocol adopted by the Department of Neuroradiology of the Azienda Ospedaliero Universitaria S. Maria della Misericordia, Udine.

In addition, for all patients, a single-shot echo-planar DTI sequence was acquired covering the whole brain (TR/TE = 8,800/74 ms; FOV= 224 mm; 54 contiguous axial slices; voxel size, 1.8 × 1.8 × 2.2 mm, b0 and b1000 s/mm2, 64 non-coplanar images). The gradient directions were uniformly distributed on a sphere. The total acquisition time for the entire protocol was ~20 min.

#### MRI Structural Data

Topographic and volumetric descriptions of the tumor were obtained by retrospectively analyzing structural imaging data routinely acquired during pre-surgery investigations.

Volumes of interest (VOIs) of patients' lesions were drawn on their T2 MRI scans using MRIcron software (https://www.nitrc.org/projects/mricron). We then normalized the Region of Interests (ROIs) to the Montreal Neurological Institute (MNI) space using the “Clinical Toolbox” (https://www.nitrc.org/projects/clinicaltbx/) for SPM8 (https://www.fil.ion.ucl.ac.uk/spm/).

The MRIcron procedure was used to overlap lesion masks (VOIs) (https://www.nitrc.org/projects/mricron). The output is a percentage overlay plot showing the percentage of overlapping lesions on a color scale.

#### Volumetric Analysis

All pre- and post-operative tumor segmentations were performed manually across axial T2-weighted and post-contrast T1-weighted MRI slices.

EOR was evaluated by using 3D T2-weighted MRI axial images as follows: (pre-operative tumor volume—post-operative tumor volume)/pre-operative tumor volume) (14).

#### DTI: Preprocessing and Fiber Tracking Analysis

Using the FSL software (Functional Magnetic Resonance Imaging of the Brain Software Library http://www.fmrib.ox.ac.uk/fsl) DTI images were pre-processed for eddy current and head movement corrections, and a brain extraction was performed using BET tool implemented in FSL.

Those pre-processed data were later analyzed using DTI-Studio, and a three-dimensional tract reconstruction of the IFOFs was created using the FACT (Fiber Assignment by Continuous Tracking) algorithm and a multiple regions of interest (ROIs) approach. We choose a FA threshold of 0.1 and a turning angle threshold of 55° to yield biologically plausible results.

In accordance with the procedures of Wakana et al. (15), for tracking the IFOF we used two ROIs both manually drawn in a coronal plane, the first ROI included the occipital lobe, the second ROI included the entire hemisphere at the anterior part of the genu of corpus callosum. For tracking the SLF the two ROIs were located on coronal planes, the first centered at the middle of the internal capsule and the second placed at the splenium of corpus callosum. These fibers clearly don't belong to the IFOF or to the SLF and should be manually removed using “NOT.”

SLF and IFOF fiber-tracking results were visually inspected to determine whether they were anatomically accurate and, if necessary, manually corrected. The number of IFOF and SLF streamlines passing through each voxel was automatically measured by the software, defined as the total NS.

The ipsilateral tumoral and healthy contralateral SLF (T_SLF_ and H_SLF_) and IFOF (T_IFOF_ and H_IFOF_) were both reconstructed and assessed for each patient to define potential differences between these white matter pathways induced by the presence of the brain tumor.

The number of streamlines (NS) was calculated in accordance with Ius et al. (8).

Specifically, for each patient, we calculated the ratio of the number of streamlines (NS-index) as follows:

- SLF NS-index= (H_SLF_–T_SLF_)/ H_SLF_;
- IFOF NS-index= (H_IFOF_–T_IFOF_)/ H_IFOF_.

### Definition of Post-operative Neurological Outcomes

Patients were examined pre-operatively, immediately after surgery, 1-week after surgery and 6 months after surgery.

Patients' outcome examination includes the presence/absence of sensory-motor deficit and speech disorders.

Regarding language deficits, two categories were established:

- Transient aphasia or dysphasia: any new language deficit owing to surgery that resolved at 6 months follow-up;

- Permanent aphasia or dysphasia: any new language deficit owing to surgery that did not resolve at 6 months follow-up.

### Statistical Analysis

Characteristics of the study population were described using the median with interquartile range of numerical variables and the percentages of categorical variables. Wilcoxon-Mann-Whitney test and non-parametric correlation were used to explore possible associations between NS-index and post-operative outcomes (expressed as dichotomous) and continuous patient characteristics, respectively.

ANOVA analysis between subjects was performed to compare patients' and healthy subjects' NS and FA for the SLF and the IFOF.

Regarding clinical parameters, post-operative language deficit was dichotomized as 0 (no deficit) and 1 (deficit: aphasia and/or dysphasia). Similarly, post-operative seizure outcome was classified as 0 (Engel Class Ia), and 1 (Engel Classes Ib-IV).

Receiver operating characteristics (ROC) analysis with estimation of 95% confidence interval (95% CI) of the area under the curve (AUC) was performed to estimate the accuracy of the NS-index in predicting post-operative outcomes, and to determine the best cut-off value to discriminate the two Engel index groups.

A bilateral *p* < 0.05 was considered significant. All statistical procedures were performed using SAS software, version 9.4 (SAS, Cary, NC, USA).

## Results

### Clinical Data

The baseline demographic, pre-operative clinical, and radiological characteristics of the study population are summarized in Table 1. Seizure was the onset symptom in 24/26 cases (92.30%). In two cases the diagnosis was incidental (7.70%). In all cases, pre-operative MRI showed hypo-intense lesions on T1-weighted sequences obtained without contrast medium and hyper-intense lesions on T2-weighted sequences. Insular lobe was involved in 17 cases, while temporal lobe in 9 cases. Immediate and 1-week post-operative language deficits were recorded in 11 cases (42.31%), while 2 patients (7.70%) developed permanent language impairment at 6 months follow-up.

**Table 1:** Baseline characteristics of study population.

**Parameters**	**Value**
**No. of patients**	**26**
**Sex**	
Male	15 (57.69%)
Female	11 (42.31%)
**Age, (years)**	
Median (years and range)	35.50 (20–64)
**Onset symptoms**	
Seizures	24 (92.30%)
Focal seizures	12 (46.15%)
Focal to bilateral tonic-clonic seizures	12 (46.15%)
No symptoms (incidental LGG)	2 (7.70%)
**Seizure Types**	
Motor	12 (46.15%)
Non-motor	12 (46.15%)
Automatism and cognitive	1 (3.85%)
Cognitive	8 (30.77%)
Sensory	2 (7.69%)
Sensory-cognitive	1 (3.85%)
**Pre-operative tumor volume (T2-weighted MRI images—cm**^**3**^**)**	
Median	36.5
Range	6–127
**EOR**	
Median	95.0
Range	50–100
**Molecular Class**	
Oligodendroglioma	6 (23.08%)
Diffuse Astrocytoma	16 (61.54%)
Astrocytoma IDH1/2 wild-type	4 (15.38%)
**Post-operative Engel class at 12 months**	
Ia	17 (65.38%)
Ib–IV	9 (34.62%)
**Post-operative neurological deficit 1 week after surgery**	
No	15 (57.69%)
Yes	11 (42.31%)
Only language	7 (26.92%)
Language + motor	4 (15.38%)
**Post-operative neurological deficit at 6 months**	
No	24 (92.30%)
Yes	2 (7.70%)
Only language	2 (7.70)
Mild	1 (3.85%)
Moderate—Severe	1 (3.85%)

*EOR, extent of surgical resection; IDH, isocitrate dehydrogenase*.

*Patients' characteristics are described using median and range for continuous variables, number of cases with relative percentages (in parentheses) for categorical variables*.

### MRI Structural Data

#### Pre-operative Tumor Volume

All the lesions were non-contrast enhancing LGGs. Pre-operative median tumor volume (cm^3^) calculated on T2-weighted MRI was 36.54 ± 23.73 (range 6–127).

#### Maximum Lesion Overlap

**Figure 1:**
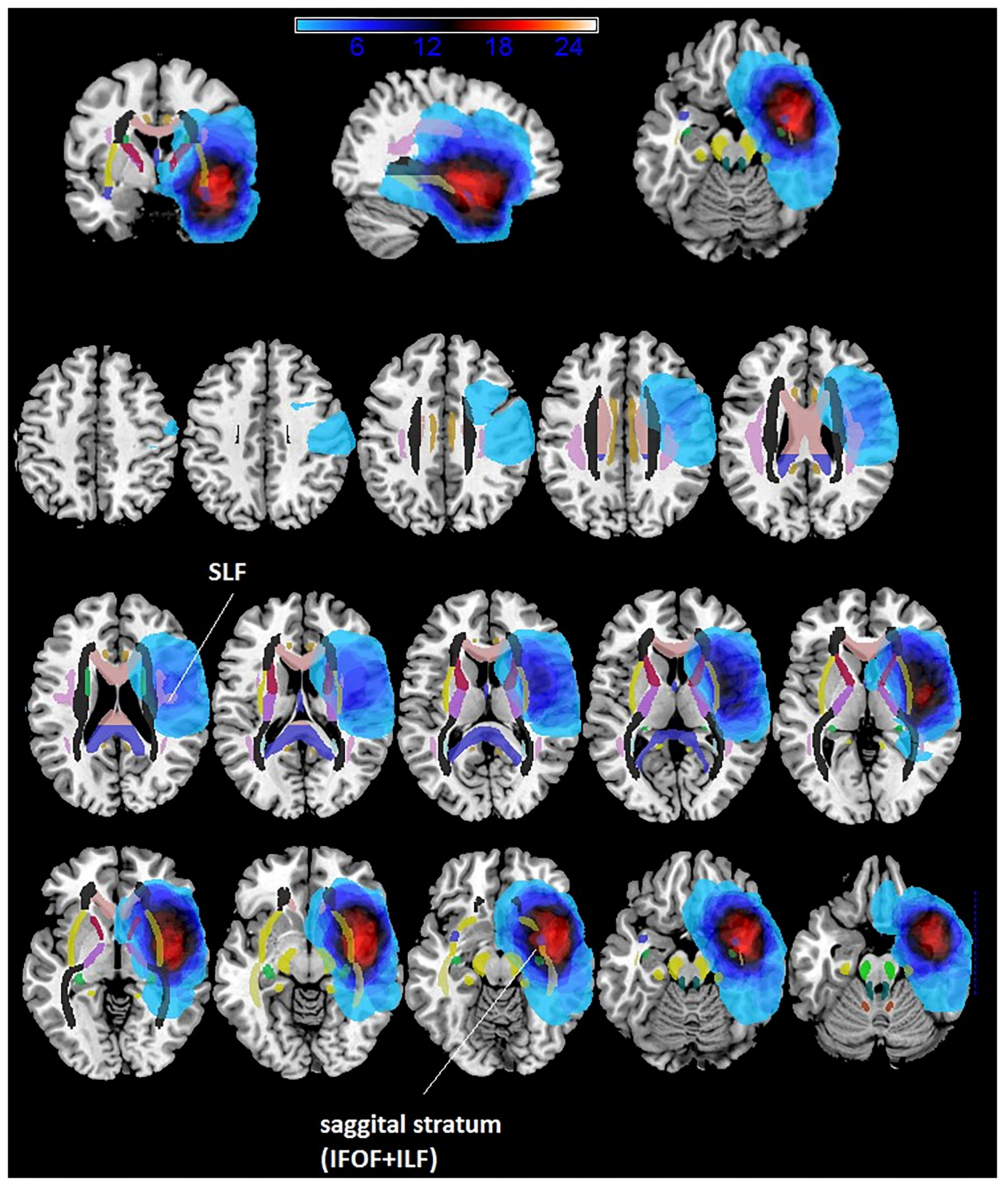
Overlaps of lesions masks of patients with temporo-insular LGGs.

The maximum overlap of the LGG lesion masks of patients mainly occurred in the left insula and superior temporal gyrus, temporal pole and the sagittal stratum (IFOF + ILF) (see Table 2 and Figure 1).

**Table 2:** Montreal Neurological Institute (MNI) coordinates of the areas showing higher % lesion overlap.

**Area**	**x**	**y**	**z**	**% overlap**
**Insula**	−39	3	−13	77%
**Sagittal stratum (IFOF+ILF)**	−39	−9	−17	73%
**Uncinate Fasciculus**	−34	2	−19	77%
**Temporal Pole**	−41	7	19	73%
**Hippocampus**	−38	−10	−17	73%
**Superior Temporal Gyrus**	−47	−5	−1	77%
**Superior Longitudinal Fasciculus**	−31	−2	18	23%
**Superior Fronto-Occipital Fasciculus**	−21	3	19	23%

*IFOF, Inferior Fronto-Occipital Fasciculus; ILF, Inferior Longitudinal Fasciculus*.

### Surgical Data

All patients were operated in Awake Surgery. In all cases, the resection was stopped according to DES and/or RTNT. The median EOR was 95% ± 12.24 (range 50–100).

### DTI Analysis and Pre-operative Risk Factors Influencing the Outcome

**Figure 2:**
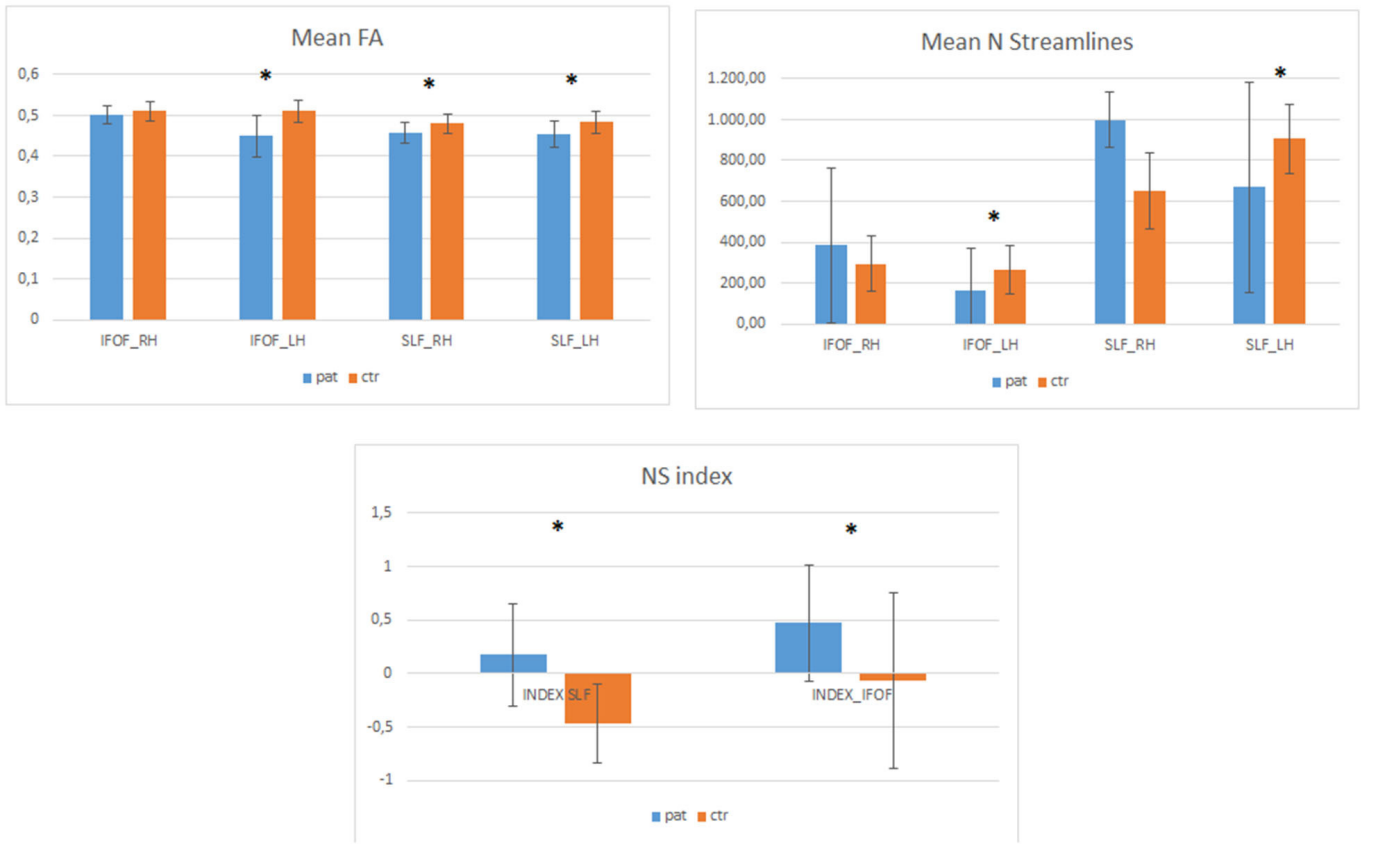
The Plot illustrates FA (fractional anisotropy) and the number of streamlines (NS) data, expressed as mean value and standard deviations, for SLF and IFOF both in healthy (right hemisphere = RH) and tumoral (left hemisphere = LH) hemispheres for the patients (pat) and healthy controls (ctr), respectively. Asterisks denote significant differences.

Data regarding FA and NS-indices of SLF and IFOF, both in healthy and tumoral hemisphere, are reported in Table 3 and Figure 2.

**Table 3:** Baseline characteristics of FA and NS-indices both for SLF and IFOF.

**DTI Parameters**	**Value**
**FA H**_**IFOF**_	
Median	0.50
Range	0.44–0.54
**FA T**_**IFOF**_	
Median	0.45
Range	0.35–0.53
**Number of streamlines in H**_**IFOF**_	
Median	299.5
Range	28–1,728
**Number of streamlines in T**_**IFOF**_	
Median	122.0
Range	2–836
**FA H**_**SLF**_	
Median	0.46
Range	0.40–0.50
**FA T**_**SLF**_	
Median	0.45
Range	0.37–0.52
**Number of streamlines in H**_**SLF**_	
Median	719
Range	199–7,521
**Number of streamlines in T**_**SLF**_	
Median	467.0
Range	142–2,494

*FA, fractional anisotropy; IFOF, Inferior Fronto-Occipital Fasciculus; SLF, Superior Longitudinal Fasciculus*.

*The characteristics are described using median and range*.

Correlation analysis between clinical-radiological data and NS- indices (SLF NS-index; IFOF NS-index) are displayed in Table 4.

**Table 4:** Correlation analysis between clinical-radiological variables and NS-indices (Spearman's Correlation).

**Spearman's correlation coefficients** ***N= 26***		
**Variables**	**IFOF NS-Index**	**SLF NS-Index**
**Age**		
Prob > |r|	−0.31417	0.05065
H0: Rho=0	0.1180	0.8059
**Pre-operative tumor volume (T2-weighted MRI images)**		
Prob > |r|	0.16393	0.17385
H0: Rho=0	0.4236	0.3957
**EOR**		
Prob > |r|	0.11995	−0.36204
H0: Rho=0	0.5595	0.0691
**FA H**_**IFOF**_		
Prob > |r|	0.13472	−0.01128
H0: Rho=0	0.5117	0.9564
**FA T**_**IFOF**_		
Prob > |r|	**−0.48632**	**−0.40253**
H0: Rho=0	**0.0118**	**0.0415**
**Number of streamlines in H**_**IFOF**_		
Prob > |r|	0.15321	0.18947
H0: Rho=0	0.4549	0.3539
**Number of streamlines in T**_**IFOF**_		
Prob > |r|	**−0.66644**	−0.23594
H0: Rho=0	**0.0002**	0.2459
**IFOF-NS Index**		
Prob > |r|	1.00000	**0.42906**
H0: Rho=0		**0.0287**
**FA H**_**SLF**_		
Prob > |r|	−0.16345	−0.12070
H0: Rho=0	0.4250	0.5570
**FA T**_**SLF**_		
Prob > |r|	−0.15800	−0.20725
H0: Rho=0	0.4408	0.3097
**Number of streamlines in H**_**SLF**_		
Prob > |r|	0.36547	0.06735
H0: Rho=0	0.0664	0.7437
**Number of streamlines in T**_**SLF**_		
Prob > |r|	−0.31966	**−0.68957**
H0: Rho=0	0.1114	**<0.0001**
**SLF-NS Index**		
Prob > |r|	**0.42906**	1.00000
H0: Rho=0	**0.0287**	
**Post-operative neurological deficit 1 week after surgery: yes vs. no**		
Prob > |r|	**−0.53457**	**−0.74217**
H0: Rho=0	**0.0049**	**<0.0001**

*Bold values indicate significant results*.

In detailed, there was a significant correlation between neurological outcome and both pre-operative SLF NS-index (*p* = 0.009, IC 95% = 0.001–0.136) and pre-operative IFOF NS-index (*p* = 0.042, IC 95% = 0.001–0.854). In addition, pre-operative SLF NS-index and pre-operative IFOF NS-index appear directly correlated to each other (*p* = 0.028; r_s_ = 0.429).

**Figure 3:**
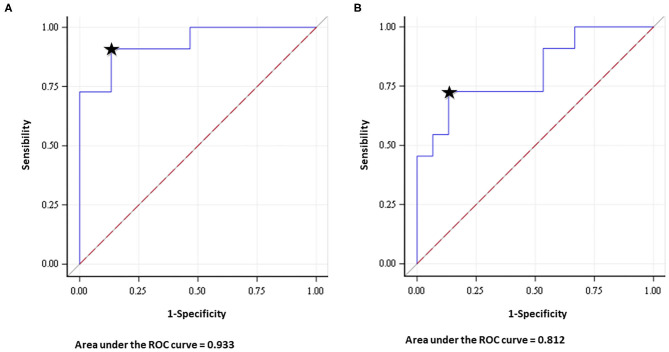
Receiver operating characteristic (ROC) analysis showed that the area under the curve of the NS (number of streamlines) index was 0.933 (CI 95% 0.836–1) and 0.812 (CI 95% 0.635–0.988) for SLF **(A)** and IFOF **(B)**, respectively. Predictive accuracy was 86.67% for SLF and 72.42% for IFOF (It is represented by the closest point to the top left corner of the graph, indicated by the black star).

We also compared patients' data with healthy controls (N = 25, all right handed, 13 F and 12 M, median age 36). The NS index was significantly different between patients and controls both for the IFOF [F _(1,49)_ = 7.64, *p* = 0.008] and the SLF [F _(1,49)_ = 29.01, *p* = 0.001]. Healthy controls data indicate that while the SLF is left lateralized (see Figure 2), the IFOF is almost bilateral. In addition, we found that patients had significantly lower number of streamlines for the left IFOF [F _(1,49)_ =4.59, *p* = 0.037] and the left SLF [F _(1,49)_ = 4.77, *p* = 0.034] when compared to controls (see Figure 2), while for the right IFOF [F_(1,49)_ = 1.23, *p* = 0.2, n.s.] and the right SLF [F_(1,49)_ = 1.6, *p* = 0.209, n.s.] they did not significantly differ (Figure 3).

In patients affected from temporo-insular LGG in dominant hemisphere, NS-index was significantly different between patients with post-operative impairment both for SLF (*p* = 0.002) and IFOF (*p* = 0.008).

In patients with post-operative deficits the median value of SLF and IFOF NS-index was 0.622 and 0.786, respectively. Otherwise in those patients with normal post-operative surgical outcome the median value of SLF and IFOF NS-index was 0.072 and 0.587, respectively.

The optimal cut-off value for the pre-operative IFOF NS-index was 0.675. It was the point with the highest sensitivity (0.727) and specificity (0.866), with a resulting area under the curve of 0.812 (CI 95% 0.635–0.988) and a predictive accuracy of 72.42%.

For the pre-operative SLF NS-index, the cut-off value of 0.248 corresponded to the point with the highest sensitivity (0.909) and specificity (0.866), with a resulting area under the curve of 0.933 (CI 95% 0.836–1) and a predictive accuracy of 86.67% (Figure 3).

**Figure 4:**
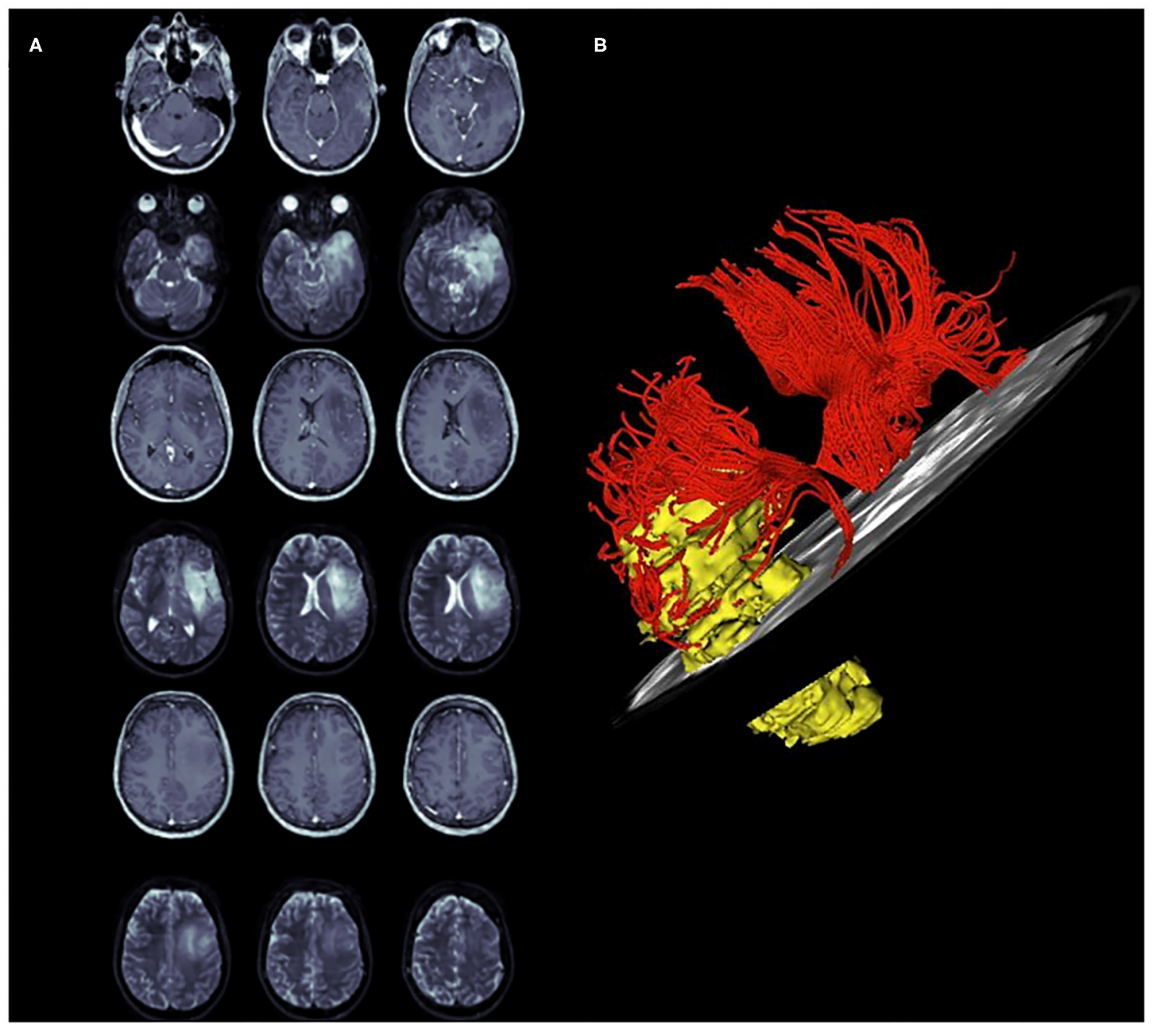
A case of left temporo-insular low-grade glioma (LGG). The pre-operative tumor volume computed on T1-weigted MRI and on T2-weighted MRI was 36 cm^3^ and 42 cm^2^, respectively (axial slices **A**). **(B)** represents a 3D neurological view of the tumor, T_SLF_ and H_SLF._ The pre-operative SLF NS-index was 0.601.

**Figure 5:**
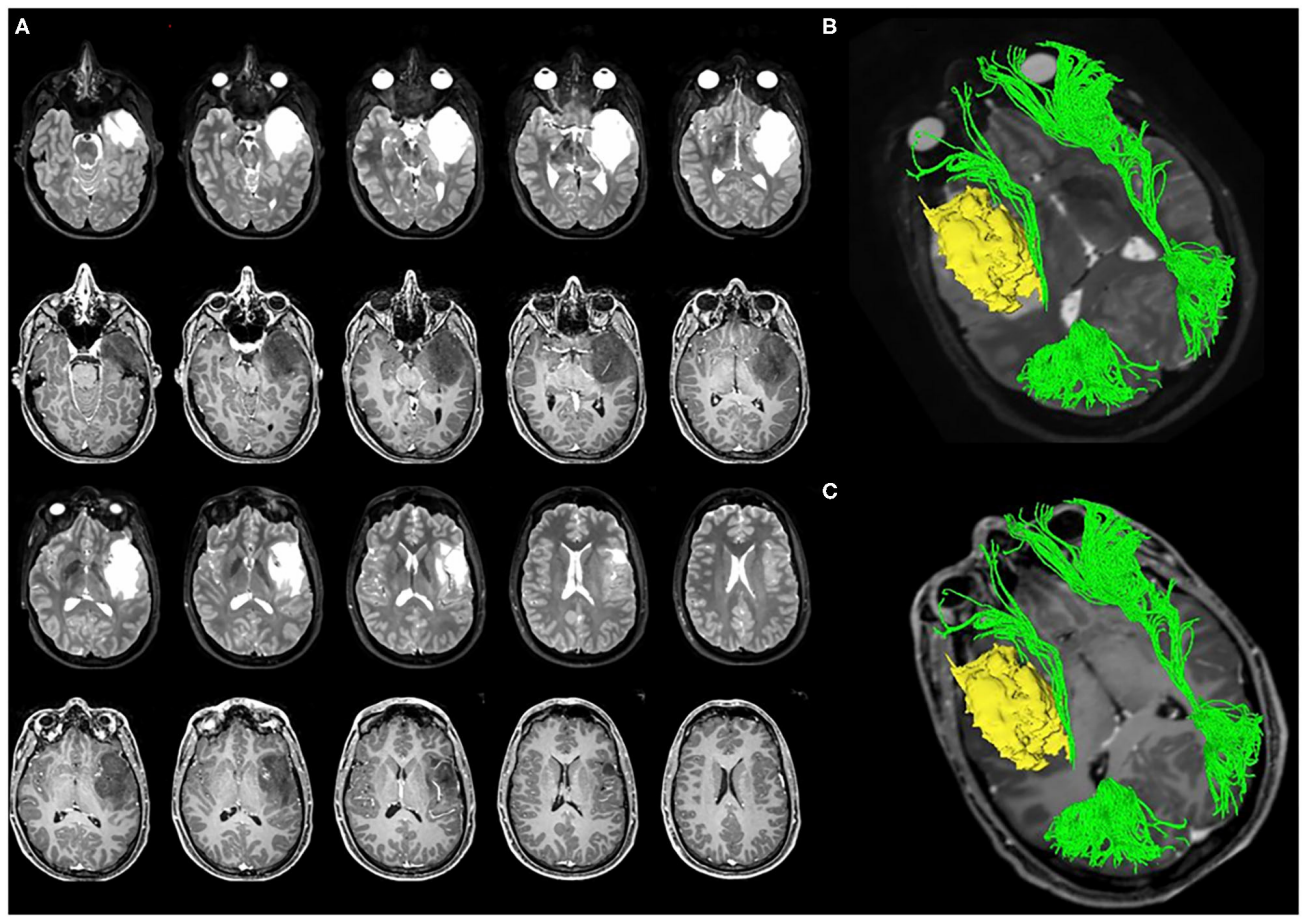
A case of left temporo-insular low-grade glioma (LGG). The pre-operative tumor volume computed on both T1-weigted MRI and on T2-weighted MRI was 38 cm^3^ (axial slices **A**). The tumor involves the Fronto-Occipital Longitudinal Fasciculus. **(B,C)** Show the 3D reconstruction of the tumor and IFOF. The pre-operative IFOF NS-index was 0.611.

Figures 4, 5 summarized two exemplificative conditions based on different values of SLF and IFOF NS-index.

The potential relationships between the structural white matter (WM) changes induced by LGG infiltrative growing and post-operative seizure outcome were also evaluated. No correlation has been highlighted between the FA, NS-indices and seizure outcome (*p* = 0.06 and *p* = 0.91, respectively for IFOF and SLF NS-indices).

A positive correlation between the pre-operative tumoral volume on T2 weighted MRI and both FA T_IFOF_ (*r* = 0.670; *p* = 0.002) and FA T_SLF_ (*r* = 0.457; *p* = 0.018).

In closing, we found a positive correlation between EOR and post-operatory status (*r* = 0.39535, *p* = 0.0456).

## Discussion

Low-grade gliomas generally affect young patients, and in almost all cases (from 70 to 90%), seizure is the onset symptom. The key point in management is based on maximal safe resection. The main technical difficulty relies on the infiltrative pattern of these tumors, especially at subcortical levels.

The cohort of patients included in this study was characterized by a relatively young age (median 35.5 years) and intermediate lesion volumes (median 36 cm^3^), which were almost completely resected reaching a high EOR (median 95%). It is therefore fundamental, especially given the young age and long-life expectancy, to develop new diagnostic strategies both for surgical planning and pre-operative estimation of surgical risks.

The substantial variability of the language network organization is recognized especially in neurosurgical literature (16, 17) and represents only a superficial part of a more complex and variable networks (14, 18–21). According to the “connectionist model,” there is a wide individual variability amongst both cortical areas and subcortical white matter. It is also well-documented that the brain is able to reorganize itself in pathological condition, especially in the clinical setting of slow growing tumors as LGG (20, 22–25).

In clinical practice, it would be of utmost importance to estimate the impact of surgery on the post-surgical outcomes, and to understand the potential functional recovery in response to an infiltrating slow growing tumor by means of objective measures (2, 26, 27). Given tumor-related plastic reshaping and reallocation of function, individual data are needed for patient counseling and risk assessment prior to surgery. Conventional MRI techniques provide purely anatomic information, resulting to be insufficient in patients with language area-related LGGs, mainly considering the higher inter-individual variance of functional language anatomy (2, 5, 7, 8, 21, 28–30). The DTI-based tractography has gradually become a well-established clinical tool with different applications, which include assessment of the subcortical pre-operative anatomy, characterization of epileptic networks and study of the connectomes (2, 21, 23, 28, 30–36).

The superior longitudinal fasciculus white matter complex involves transmission of speech, forming the dorsal pathway within the dual-stream model of language processing (37–39). The IFOF is mainly involved in language, specifically in lexical and semantic processing (40). With respect to naming abilities, a study involving 99 patients with glioma showed that noun naming deficits depended on damage to parts of the sagittal stratum (including the inferior fronto-occipital fasciculus and the inferior longitudinal fasciculus), in addition to cortical lesions (41). Moreover, the IFOF, together with the inferior longitudinal fasciculus, is the neuroanatomical correlate of the ventral reading route (42–45). Concerning reading, we reported that surface dyslexia could be due to impaired ventral/lexical route (as evidenced by a fractional anisotropy decrease along the inferior fronto-occipital fasciculus) in patients with glioma (46). The role of the IFOF in reading and in surface dyslexia, and of the SLF in phonological dyslexia was confirmed on a larger group study in which pre-, intra- and post-surgery reading data were presented (47).

In this investigation, we used quantitative DTI analysis in patients with LGGs involving the SLF and IFOF to estimate the potential predictive role of this technique in terms of functional post-operative outcomes. Two different white matter parameters were considered: FA and NS.

Current literature has widely documented that the FA value is lower in patients affected by glioma in comparison to FA values in healthy subjects. Our investigation confirmed these overall results. Furthermore, we found that FA values in T_SLF_ and T_IFOF_ were smaller than those in H_SLF_ and H_IFOF_, confirming the slow infiltrative growing of LGGs (48–51).

All patients were without neurological impairment at diagnosis, despite the tumoral infiltration of white matter, supporting the brain capability to reorganize itself in pathological condition (22–24). The comparison of tumoral patient data with healthy controls highlighted that the significant effects found on the NS index, both for the IFOF and the SLF, indicate a decreased white matter representation in the pathological left hemisphere. For the right hemisphere, the differences between patients and controls were not significant, however, the mean number of streamline and standard deviations are suggestive of higher values for patients, thus implying possible plasticity related reshaping of the right hemisphere. The integrity of the IFOF and SLF, known to be part of the language-related network, were then investigated. When comparing the DTI differences between the H and T hemispheres, two indices were elaborated (pre-operative SLF NS-index and pre-operative IFOF NS-index) and correlated to post-operative outcomes. Our study found that patients with pre-operative indices close to 0 (that implies a similar number of streamlines in both hemispheres) generally had less probability of developing post-operative neurological impairment. Patients with pre-operative indices close to 1 (that means a low number of streamlines in tumoral hemisphere), in contrast, were more likely to develop post-operative deficits. An SFL NS-index <0.248, and an IFOF NS-index <0.675 have been associated with better post-operative clinical outcomes. These results are consistent with a previous study based on DTI analysis of cortico-spinal tract in patients with premotor LGGs (8), confirming that the NS analysis of the main subcortical pathways is a valid and promising approach to pre-operatively estimate the risk of post-operative deficits.

A positive correlation was found between pre-operative SLF NS-index and pre-operative IFOF NS-index, suggesting that lesions infiltrated both, which is also consistent with the MRI structural data analyses. Lesions were shown to be mainly localized in the insula and superior temporal lobes, which are areas crossed by both the IFOF in their inferior portion and the SLF in the upper portion.

The correlations between the pre-operative tumoral volume on T2 weighted MRI and both FA T_IFOF_ and FA T_SLF_ suggest that higher pre-operative tumor volume may induce a higher white matter infiltration probability.

In closing, we found a positive correlation between EOR and post-operatory status, thus suggesting that maximizing the resection implies assuming the risk of observing a transient immediate post-surgery decrease. In the study, we also investigated the potential relationships between the structural changes of white matter tracts, induced by LGG infiltrative growing, and post-operative seizure outcomes. No correlation was found between the pre-operative SLF NS-index, pre-operative IFOF NS-index and post-operative seizure outcomes. These findings could be based on the possibility that the epileptogenic foci were located outside of the SLF and IFOF in our sample. It is therefore possible that concerning the cases of seizure-free patients within our cohort, the epileptogenic foci could have likely been removed with the tumor and peritumoral cortex. With regards to the patients showing poor post-operative seizure control, the failure or the impossibility of removing the epileptogenic zone, and the presence of a possible secondary epileptic focus need to be considered. In both cases, IFOF and SLF infiltration may not have been relevant. Epileptogenic processes in tumor-related epilepsy (TRE) are multifactorial and not fully understood (52). Increased evidence suggests that, in up to two-thirds of patients with glioma, seizures arise from the peritumoral cortex, due to induced changes in neurotransmitters, environment and electrical properties of these regions (53, 54). The likelihood of seizure control is thus related to the possibility of resection of the epileptogenic zone (EZ), nested in the peritumoral tissue. For this reason, the extent of resection in now recognized as one of the strongest prognostic factors for post-operative seizure control (1, 55, 56). We acknowledge that our sample is small to draw definitive considerations. Moreover, aphasic seizures were poorly represented. Regarding WM tracts, the clinical challenge, in primary brain tumor epilepsy, is represented not only by the determination of the EZ, but also by the identification of the underlying epileptic network, which requires a complex neurophysiological presurgical work-up.

There are several limitations of our study. The most important is the retrospective nature of the investigation and the small simple size. Concerning the DTI methodology, different tracking approaches exist, although there is no consensus yet on the most suitable one. It would be interest to verify these preliminary results by using other DTI approaches.

In addition, this approach is only applicable to LGGs due to their specific biologically determined growth pattern (57). The elaboration of NS-indices based on numbers of streamlines in both hemispheres, strengthens the results, limiting the bias due to the individual variability. Moreover, an extensive neuropsychological assessment has not been included in this study, which has, however, been considered in our ongoing future studies currently underway. Despite the inherent limitation of DTI analysis, which include data acquisition, bio-mathematical models, user dependency, and software programs (58–60), it still represents the only way to investigate white matter in humans *in vivo* (7). Overall, we recognize that these preliminary results require a further validation increasing the study population and planning future prospective studies.

Future longitudinal studies, based on comparison between structural WM in healthy and tumoral hemisphere should be developed to address the strongest predictive measures of surgical risk for lesion infiltrating the functional subcortical pathways. The integration of this approach with an extensive pre- and post-operative neuropsychological assessment might provide insights into compensatory mechanisms for language deficits on the level of white matter plasticity, in patients with brain tumors. It could be of clinical importance to assess the ability of the proposed indices in identifying the risk of permanent deficits in a larger cohort of patients.

## Conclusion

These preliminary results highlight the potential role of pre-operative DTI in assessing the risk of post-operative transient language impairment in patients undergoing surgical resection of LGGs involving the AF and IFOF in dominant hemisphere. This analytical pre-operative approach may represent a feasible predictive tool for patient counseling and risk assessment prior to surgery and pave the way to standardize approaches in glioma surgical management.

## Data Availability Statement

The raw data supporting the conclusions of this article will be made available by the authors, without undue reservation.

## Ethics Statement

The studies involving human participants were reviewed and approved by the local Ethics Committee, Comitato Etico Unico Regionale del Friuli Venezia Giulia, approved this investigation (protocol N.0036567/P/GEN/EGAS, ID study 2540). Written informed consent was not provided because considering the retrospective nature of the study, written consent to participate in the study was not applicable. Written informed consent was obtained for surgery.

## Author Contributions

TI and BT: conception and design. IG, CB, and AN: acquisition of data. FP: formal analysis. FP and MM: software. TI, BT, TS, GP, and MS: supervision. TI, BT, and MS: validation. TI, BT, and GP: writing—original draft and review and editing. All authors have read and agreed to the published version of the manuscript. All authors had full access to all the data in the study and take responsibility for the integrity of the data and the accuracy of the data analysis.

## Conflict of Interest

The authors declare that the research was conducted in the absence of any commercial or financial relationships that could be construed as a potential conflict of interest.

## Abbreviations

AF: arcuate fasciculus
AS: awake surgery
ASM: anti-seizure medication
AUC: area under the curve
DES: direct electrical stimulation
DTI: diffusion tensor imaging
EOR: extent of resection
EZ: epileptogenic zone
FA: fractional anisotropy
FACT: fiber assignment by continuous tracking
FSL: Functional Magnetic Resonance Imaging of the Brain Software Libreary
H_SLF_: healthy SLF
H_IFOF_: healthy IFOF
IFOF: inferior fronto-occipital fasciculus
ILAE: International League Against Epilepsy
LGG: low-grade glioma
MNI: Montreal Neurological Institute
MRI: magnetic resonance imaging
NS: number of streamlines
ROC: receiver operating characteristics
ROIs: region of interests
RTNT: real time neuropsychological testing
SLF: superior longitudinal fasciculus
T_SLF_: tumoral SLF
T_IFOF_: tumoral IFOF
TRE: tumor-related epilepsy
VOIs: volumes of interests
WHO: World Health Organization
WM: white matter.
